# Accelerating Corrosion of Pure Magnesium Co-implanted with Titanium *in Vivo*

**DOI:** 10.1038/srep41924

**Published:** 2017-02-07

**Authors:** Peng Hou, Pei Han, Changli Zhao, Hongliu Wu, Jiahua Ni, Shaoxiang Zhang, Jingyi Liu, Yuanzhuang Zhang, Haidong Xu, Pengfei Cheng, Shen Liu, Yufeng Zheng, Xiaonong Zhang, Yimin Chai

**Affiliations:** 1 Orthopaedic Department, Shanghai Jiao Tong University Affiliated Sixth People’s Hospital, Shanghai 200233, China; 2 State Key Laboratory of Metal Matrix Composites, School of Materials Science and Engineering, Shanghai Jiao Tong University, Shanghai 200240, China; 3 Suzhou Origin Medical Technology Co. Ltd., Suzhou 215513, China; 4 Department of Materials Science and Engineering, College of Engineering, Peking University, Beijing 100871, China

## Abstract

Magnesium is a type of reactive metal, and is susceptible to galvanic corrosion. In the present study, the impact of coexistence of Ti on the corrosion behavior of high purity Mg (HP Mg) was investigated both *in vitro* and *in vivo*. Increased corrosion rate of HP Mg was demonstrated when Mg and Ti discs were not in contact. The *in vivo* experiments further confirmed accelerating corrosion of HP Mg screws when they were co-implanted with Ti screws into Sprague-Dawley rats’ femur, spacing 5 and 10 mm. Micro CT scan and 3D reconstruction revealed severe corrosion morphology of HP Mg screws. The calculated volume loss was much higher for the HP Mg screw co-implanted with Ti screw as compared to that co-implanted with another Mg screw. Consequently, less new bone tissue ingrowth and lower pullout force were found in the former group. It is hypothesized that the abundant blood vessels on the periosteum act as wires to connect the Mg and Ti screws and form a galvanic-like cell, accelerating the corrosion of Mg. Therefore, a certain distance is critical to maintain the mechanical and biological property of Mg when it is co-implanted with Ti.

Magnesium and its alloys have gained attention in recent decades as metallic biomaterials due to their excellent biocompatibility, mechanical properties and biodegradability^1^,^2^,^3^. Numerous studies have focused on the orthopedic applications of Mg because its elastic modulus is similar to that of bone, which minimizes the stress shield effect^4^,^5^. In addition, the released Mg^2+^ ions can stimulate new bone formation^6^,^7^. Mg-based bone screws, plates, and intramedullary nailing systems have been proven to be capable as degradable implants^8^,^9^,^10^. Currently, several pilot clinical trials have been performed. Windhagen *et al*.^11^ demonstrated that degradable MgYREZr screws did not cause foreign body reaction, osteolysis, or systemic inflammatory reaction and were equivalent to titanium screws for the treatment of mild hallux valgus deformities. Zhao *et al*.^12^ fixed vascularized bone graft with high purity Mg screws in patients with osteonecrosis of the femoral head and found that Mg screws provided promising bone screw fixation and presented considerable potential for medical applications.

In the clinic, different metallic biomaterials might be co-implanted to maximize the therapeutic effect. For example, co-implanted Ti-6A1-4V and Co-Cr alloys have been used in total hip arthroplasty (THA) since the 1990s^13^. And in dentistry, Ti materials are often selected as endo-osseous implants with other alloys served as suprastructure^14^. This could also occur to Mg biomaterials in orthopedic applications. In a recent clinical study, Yu *et al*.^15^ used vascularized iliac grafting, together with commercial cannulated compression screws and magnesium screws to treat displaced femoral neck fractures in young adults. However, it is well known that the corrosion behavior of Mg would be changed if it is in contact with other metal, or with the β-phase in Mg alloy^16^. The accelerated corrosion rate of Mg would result in loss of mechanical properties and even failure of the orthopedic implants. For example, a few years after the Ti-6A1-4V and Co-Cr alloys were used in THA, some researchers observed significant corrosion in the head-neck taper region^17^,^18^. Other laboratory experiments drew the conclusion that Ti-6A1-4V, Co-Cr-Mo coupled with stainless steel can be regarded as clinically unsafe^19^.

Mg and its alloys are especially susceptible to galvanic corrosion because of their inferior ability to form a compact oxide on surface^20^. In an earlier study, Lambotte^21^ reported a case in which an iron wire cerclage at the fibula and Mg disc with six steel screws were inserted at the tibia. It was observed that one day after the operation, the patient experienced extensive subcutaneous gas cavities, local swelling and pain. Although it is well known that the corrosion rate and corrosion behavior of Mg are fatal to the implantation, unfortunately, few studies have addressed the change of the corrosion rate and corrosion behavior of Mg under conditions in which Mg and another metal were co-implanted *in vivo*. Herein, the aim of this study is to investigate the effect of titanium screws co-implantation on the corrosion behavior of pure magnesium, as well as its impact on the osteogenesis.

## Materials and Methods

### Materials preparation

The extruded high-purity Mg (HP Mg, more than 99.98 wt.%; 0.002 wt.% Si; 0.0015 wt.% Fe; 0.0008 wt.% Al; 0.0008 wt.% Mn; 0.0002 wt.% Ni; 0.0003 wt.% Cu) and Ti (TA1ELI, 99.8 wt.%) used in these experiments were supplied by Suzhou Origin Medical Technology Co. Ltd., China. The HP Mg and Ti disc samples with a diameter of 7.5 mm and a thickness of 1 mm were used in the immersion experiments *in vitro*. The discs were ground with SiC paper up to 1200 grit followed by ultrasonically rinsing with 100% ethyl alcohol. In the *in vivo* experiments, the HP Mg and Ti screws with an outer diameter of 2.0 mm, inner diameter of 1.6 mm, screw pitch of 0.6 mm and length of 10.0 mm were prepared. The screws were sterilized with 25 kGy of ^60^Co radiation.

### Immersion test

HP Mg disc was fixed on the plastic mold, and Ti disc was fixed on another mold at a distance of 5 or 10 mm. Then, the mold was immersed in 250 ml of phosphate buffered saline (PBS, prepared as described by Lewis AC *et al*.^22^) at 37 °C. The HP Mg and Ti discs directly connected with the copper wire were designated as Group 0, while Group 5 and Group 10 represents the HP Mg and Ti discs fixed at a distance of 5 and 10 mm, respectively. The two HP Mg discs that were fixed at a distance of 10 mm were used as the control group. After 1 week of immersion, the samples were removed from PBS and ultrasonically rinsed with 180 g/L chromic acid and a 10 g/L AgNO_3_ solution followed by distilled water and were dried with air flow. Surface morphology was analyzed by scanning electron microscopy (SEM, JEOL 7600). The samples were weighed and the weight loss rate (R_WL_) was calculated as per formula (Eq. 1).


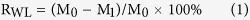


where M_0_ is the initial mass of the HP Mg samples; M_1_ is the mass after immersion.

### Surgical procedure

All animal experiments were authorized according to the Guidance Suggestions for the Care and Use of Laboratory Animals (issued by the Ministry of Science and Technology of the People’s Republic of China) and were approved by the Animal Care and Experiment Committee of Sixth People’s Hospital affiliated to Shanghai Jiao Tong University, School of Medicine. Seventy-two male 4-month Sprague Dawley rats with an average weight of 286 g (240–328 g) were used. All rats were anesthetized by 3% pentobarbital sodium (0.1 ml/100 g body weight). Surgical site was sterilized with povidone iodine, and the left leg was shaved and exposed via the anterolateral approach. Two parallel transcortical implantation beds with a diameter of 1.8 mm were pre-drilled separately on femoral diaphysis, with a spacing of 5 or 10 mm. Then, the HP Mg screws were implanted at the distal end of femur after countersinking with a drill bit tap. In the experimental group, a Ti screw was implanted at the proximal end. The Ti screw and HP Mg screw with a spacing of 5 or 10 mm was named MT 5 and MT 10, respectively. In the control group, another HP Mg screw was implanted at the proximal end. The two HP Mg screws with a spacing of 5 or 10 mm were named MM 5 and MM 10, respectively.

All implants were tolerated by the rats, and no antibiotics were given. The rats had normal activity, and no infections were observed post operation.

### Micro-CT scan

The rats were sacrificed at 2, 4, and 8 weeks post-operation. Micro-CT scan was conducted using a Laboratory Micro-CT Scanner eXplore RS 80 (GE Healthcare, Little Chalfont, UK). The X-ray tube was set at 80 kV and 450 μA with a scan resolution of 45 μm and exposure time of 400 ms. 3-D reconstruction of the HP Mg screws was conducted via Micro View 2.2 Advanced Bone Analysis Application software (GE Health Systems, Waukesha, WI, USA). The volume of the remaining HP Mg screws was measured, and the volume loss ratio (R_VL_) was calculated as per formula (Eq. 2).


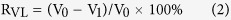


where V_0_ is the initial screw volume, and V_1_ is the residual screw volume.

### Pullout test

Pullout test was performed by Material Testing Machine (Shanghai Baihe Instrument Technology Co. Ltd., China). The max load was recorded.

### Histological test

The rat femurs were collected at 2, 4 and 8 weeks post-operation; fixed in 4% formalin for 3 days; and were then dehydrated in graded ethanol followed by methyl methacrylate embedding. A low speed precision cutting machine (DTQ-5, HOVKOX, China) was used to perform lengthways sectioning parallel to the longitudinal axis of both the femur and screws. Sections were reduced to a thickness of 90 μm by an EXAKT micro-grinder system (EXAKT, Germany). The sections were stained with toluidine blue, and histological images were recorded by optical microscopy (Leica DM2500, Leica, Germany). The bone- implant contact (BIC) was carried out by optical microscopy equipped with image analyzer (Image-Pro Plus, Media Cyberbetics, USA). The percentage of BIC was calculated as per formula (Eq. 3).


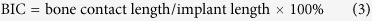


### Statistical analysis

The data are expressed as the means ± standard deviations. Statistical analysis was performed with SPSS (SPSS 17.0 Inc., Chicago, USA). One-way ANOVA and Student-Newman-Keuls post hoc tests were used to determine the level of significance. p values less than 0.05 were considered to be significant, and p values less than 0.01 were considered to be highly significant.

## Results

### *In vitro* corrosion behavior of HP Mg affected by Ti

**Figure 1:**
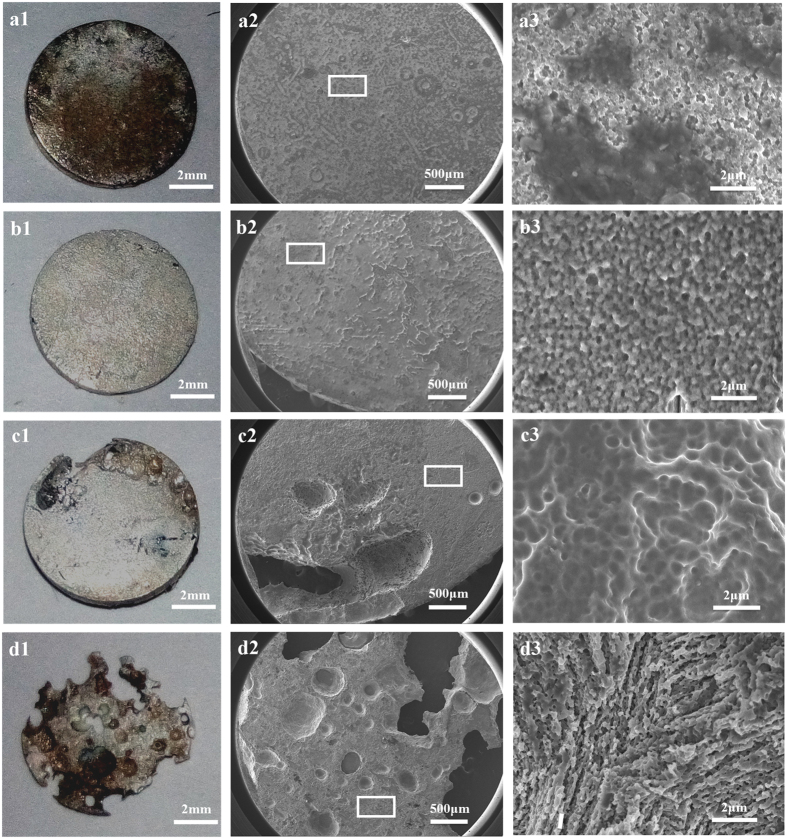
Gross observations (a1–d1) and SEM morphologies (a2–d2 and a3–d3) of the HP Mg discs. (**a**) control group; (**b**) Group 10; (**c**) Group 5; and (**d**) Group 0.

Gross observation in Fig. 1 shows that the HP Mg discs in the control group remained integrated after 1 week of immersion. The SEM morphologies revealed that the samples experienced relatively uniform corrosion (Fig. 1a2). Only small pits could be seen on the corrosion surface. In contrast, the HP Mg discs in Group 0 suffered severe corrosion and were almost depleted. Due to the great potential difference, which is −1.6 V vs SCE for pure Mg^23^ and −0.4 V vs SCE for Ti in PBS^24^, galvanic corrosion occurred and significantly accelerated the corrosion of Mg. The SEM results demonstrated that there was a large area that had non-uniform degradation (Fig. 1d2).

**Figure 2:**
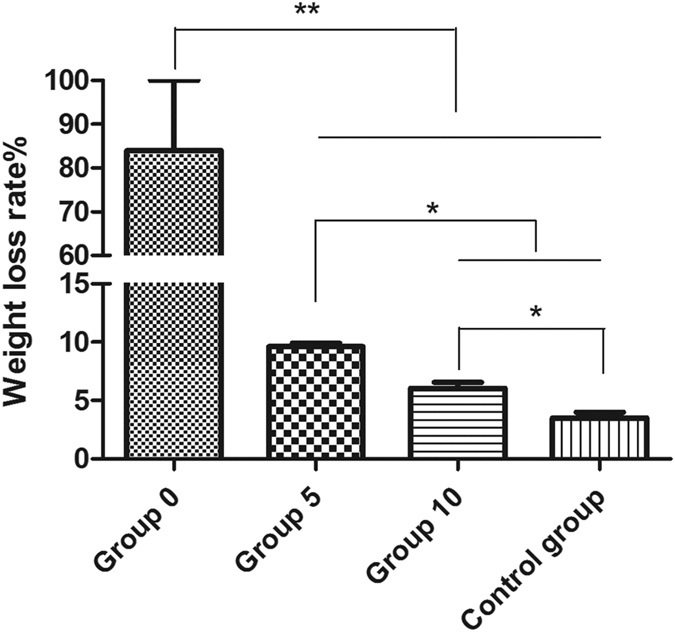
Weight loss rate of HP Mg disc. *p < 0.05; **p < 0.01.

It was observed that enhanced corrosion occurred in the HP Mg samples in Group 5 when the Mg and Ti disc were not in contact with each other, i.e., the galvanic corrosion unit did not formed. Several large corrosion pits could be seen on the edge of HP Mg disc according to the SEM morphology (Fig. 1c2). When the distance increased to 10 mm, the surface of HP Mg disc became relatively flat with many small corrosion pits (Fig. 1b2). Accordingly, a significantly difference (p < 0.05) was revealed in the weight loss rate between Group 5 (9.6 ± 0.4%, n = 3) and Group 10 (6.0 ± 0.6%, n = 3). Moreover, as illustrated in Fig. 2, the weight loss rate of both Group 5 and Group 10 are obviously higher than the Control group (3.5 ± 0.7%, n = 3; p < 0.01 and p < 0.05 respectively).

### Micro-CT scan

**Figure 3:**
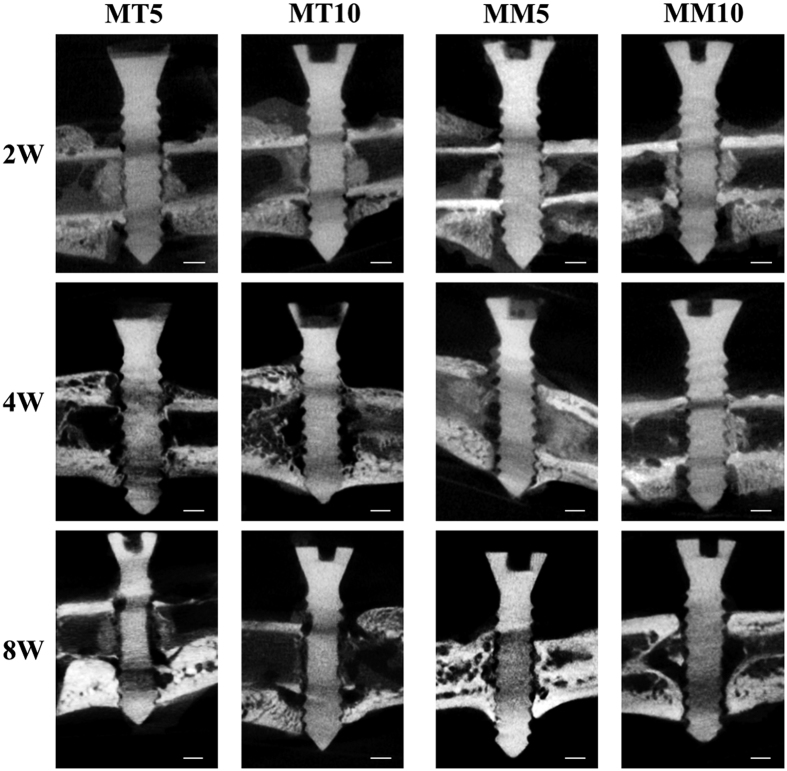
CT image of HP Mg screws in different groups. The bar represents 1 mm.

**Figure 4:**
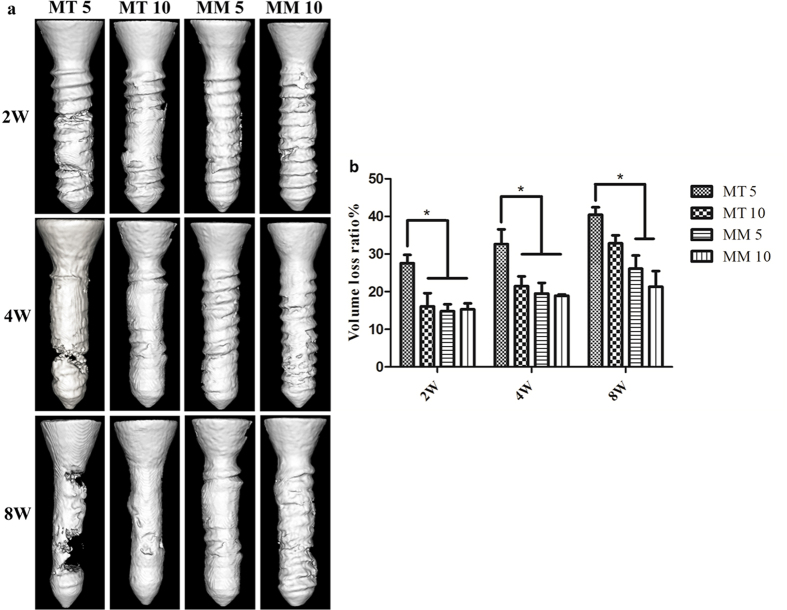
3D reconstruction (**a**) and volume loss percentage (**b**) of HP Mg screws in different groups. *p < 0.05.

The representative 2D micro CT images of femurs with screws are shown in Fig. 3. It is demonstrated that a region of low density locates around the cortical bone in all groups during the 8 weeks of implantation. In group MT 5 and MT 10, a massive cavity was found around the screws after 4 weeks of implantation and the screws showed a blurring screw thread contour and a thin screw body after 8 weeks of implantation. Newly formed bone was found around the HP Mg screws, but the connection was not tight. In contrast, the HP Mg screws in both Group MM 5 and MM 10 remained relatively integrated, with only mild changes in the depth of the screw thread after 8 weeks implantation. A large amount of new bone formation that had a tight connection with HP Mg screws was observed. No obvious difference of screws degradation or osteogenesis was found between Group MT 5 and MT 10 at each time point. However, the 3D reconstruction of the HP Mg screws shown in Fig. 4a illustrates that the HP Mg screws in Group MT 5 have a higher corrosion rate than that in Group MT 10. A corrosion crack was found on the HP Mg screws as early as 4 weeks in Group MT 5, and a wider crack was found after 8 weeks of implantation, while the HP Mg screws in Group MT 10 remained integrated after 8 weeks. Although no obvious crack was found in Group MT 10, the screw had a thinner body than that of Group MM 5 and MM 10. The calculated volume loss (Fig. 4b) confirmed that during the 8 weeks of implantation, the volume of the Mg screws in Group MT 5 reduced more than those in Group MM 5 and MM 10. No significant difference was observed between Group MM 5 and MM 10.

### Histological analysis

**Figure 5:**
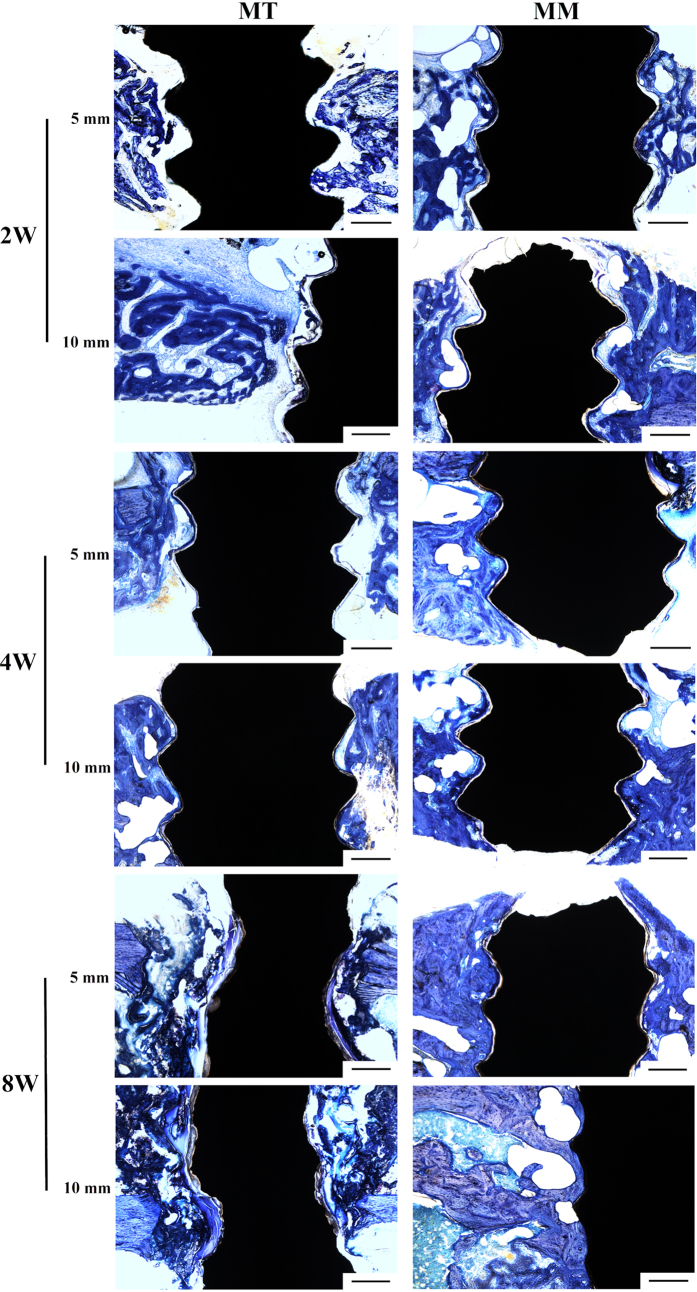
Histological analysis of the bone tissue around the magnesium implant. The bar represents 400 μm.

**Figure 6:**
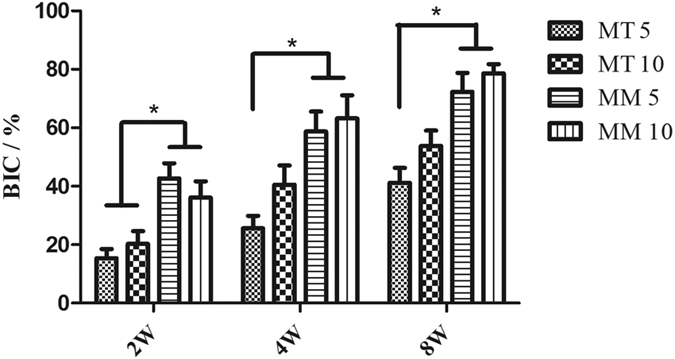
Calculated bone-implant contact of HP Mg screws in different groups. *p < 0.05.

Figure 5 illustrates the results of a histological analysis of the bone tissue surrounding the Mg screws. In Group MT 5 and MT 10, inadequate osteogenesis was found after 2 weeks of implantation. Massive cavities were observed in the cortical bone and around the screws, and only part of the newly formed bone (stained in dark blue) contacted the screw. After 4 weeks of implantation, cavities still existed around the screws and the bone tissue did not directly contact the screws; instead, a gap that contained an osteoid structure (stained in light blue without cell structure) existed between the screw and cortical bone. Although fewer cavities were observed after 8 weeks of implantation, the gap between the screw and cortical bone was wider and the content in this gap became disordered. Additionally, it was obvious that the screws in Group MT 5 and MT 10 degraded more severely than those in Group MM5 and MM 10. In contrast, although inadequate osteogenesis and cavities also existed after 2 weeks of implantation in Group MM5 and MM 10, the newly formed bone tissue contacted HP Mg screws well after 4 and 8 weeks of implantation. The BIC presented in Fig. 6 indicates poor osseointegration in Group MT 5. When the distance between Mg and Ti screw increase to 10 mm, the BIC increase slightly. No statistical difference was found between Group MM 5 and MM 10.

### Pullout test

**Figure 7:**
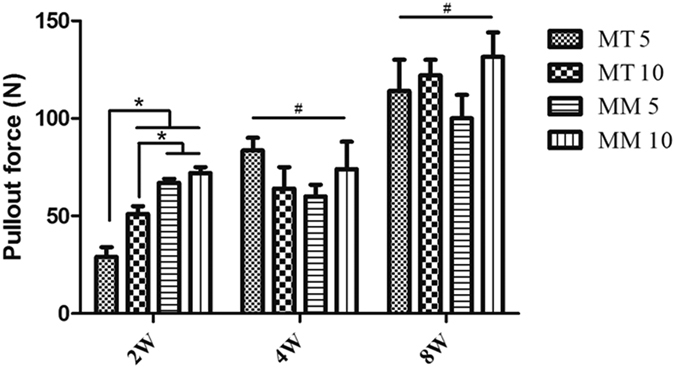
Pullout force of different groups. *p < 0.05; ^#^p > 0.05.

A dramatic decrease of the pullout force of the HP Mg screws was observed in the first 2 weeks of implantation in Group MT 5 and MT 10. However, no significant difference was found after 4 weeks of implantation among all groups. In general, the pullout force increased steadily over the duration of implantation (Fig. 7). No statistical difference was found between Group MM 5 and MM 10.

## Discussion

The corrosion behavior of Mg is one of the crucial factors that should be taken into consideration when it is used as orthopedic implants. Fast degradation of Mg implants decreases the mechanical stability and osseointegration ability. Co-implantation of Mg with other metallic materials is a possible situation that occurs in future clinic. Therefore, the changes in the corrosion behavior of Mg affected by other metals should not be neglected.

Mg is a type of reactive metal. A galvanic cell will formed if Mg and other biomedical metal are in contact, in which Mg acts as the anode and the other metal acts as the cathode. As a result, the corrosion rate of Mg will increase. The hazard of galvanic corrosion has long been noted both in industry^25^,^26^ and in the clinic^21^,^27^,^28^,^29^. Therefore, direct contact of Mg and other metals should be avoided in clinical use.

More importantly, the *in vitro* immersion test demonstrated increased corrosion rate of Mg when Ti was not even in contact with Mg. In general, the corrosion of magnesium includes self-corrosion and galvanic corrosion. It was reported that during galvanic corrosion, the surface morphology was dramatically different from the filiform structures associated with free corrosion^30^. In this study, the SEM demonstrated the change of surface morphology, indicating the galvanic-like corrosion.

The *in vivo* experiments also revealed enhanced corrosion rate of Mg screw when co-implanted with Ti screw. The Mg screws in Group MT 5 suffered the most severe corrosion in morphologies and the volume loss was significantly higher than those of the Group MM 5 and Group MM 10. With the increasing distance between Mg and Ti screws, the Mg screws in Group MT 10 exhibited no difference in volume loss from Group MM 5 or Group MM 10. In addition, the representative 2D micro CT images of a femur with screws and the 3D reconstruction showed the most severe corrosion site was the junction of the cortical bone and the screw. One of the probable reasons is that the peak load of the implant is in this area^31^, which might lead to reduced mechanical strength and accelerated degradation^32^,^33^. On the other hand, the volume loss test clearly showed faster degradation in Group MT 5 than that of Group MM 5 and MM 10, indicating that the co-implanted Ti screw might also be contributed to the accelerating corrosion.

**Figure 8:**
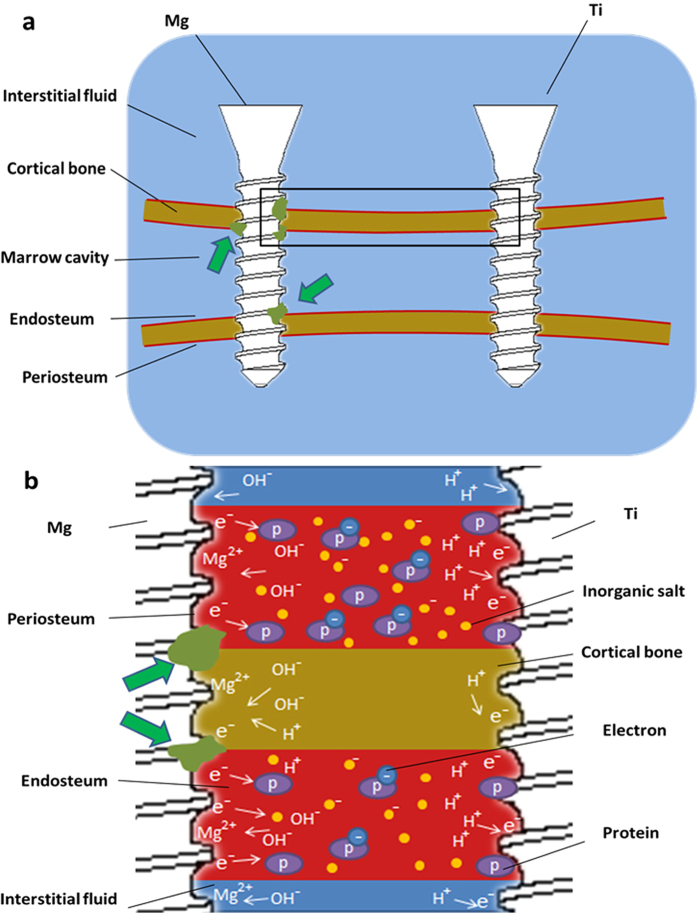
(**a**) Schematic diagram of the femoral diaphysis with screws implanted. The green arrows point the site that is most susceptible to corrosion. (**b**) Possible corrosion mechanism of HP Mg screws when co-implanted with Ti screw. (**b**) is the magnification of rectangular region in (**a**).

Figure 8 is the schematic diagram of the femoral diaphysis with screws implanted. According to the result of micro CT, the most severe corrosion site was stained in green in Fig. 8a. It should be noticed that these sites were in contact with periosteum or endosteum, which contain abundant blood vessels. It is well known that the plasma contains various proteins, some of which could play a role in the electron transport^34^. Chen *et al*.^35^ found side chains of four aromatic amino acids (Phe, His, Tyr, and Trp residues) may promote methionine and cystine residues to participate in the protein electron hole transport. The electrical conductivity of blood vessels was also reported in many researches^36^,^37^,^38^. Thus, as depicted in Fig. 8b, it is hypothesized that electrons at anode might migrate to the cathode (Ti screw) through binding with proteins. The electrically conductive blood vessels connected Mg and Ti screws, together with body fluid, formed a galvanic-like cell. The degradation of Mg also occurred in the other part of Mg screw, however, due to limited electron transfer, the corrosion rate was relatively slow. Moreover, as the distance between Mg and Ti screws increases, the electric resistance will increase proportionally and the effect of the potential difference will diminish. It should be pointed out that tissues such as bone and muscle etc. have been proved to have electric resistivity^39^, indicating that tissues other than blood vessels might also be involved in the process of electron transport. However, exact mechanism needs to be further investigated.

Histological results indicated less new bone tissue ingrowth and indirect bone-implant contact in Group MT 5 and MT 10 compared with those in Group MM 5 and MM 10. Fast corrosion of Mg results in high local Mg^2+^ concentration and alkalization, which is unfavorable to bone cell proliferation and osseointegration^40^,^41^,^42^. Besides, a significant decrease in pullout force was observed in Group MT 5 after 2 and 8 weeks of implantation. As rigid internal fixation is needed for fracture healing, the accelerated corrosion process of the HP Mg screws in Group MT 5 might not meet the clinical requirements.

It is noticed that the effect of Ti screws on the corrosion behavior of HP Mg screws decreases as their spacing increases. Hence, it is suggested that there might be a “sphere of influence” around Mg when it is implanted *in vivo*. If other metal such as Ti is implanted in this sphere, the corrosion rate of Mg will be accelerated, otherwise, the galvanic-like corrosion will have slight impact on Mg. Therefore, the potential risk of accelerating corrosion when Mg and other metals are co-implanted should be noted in clinic.

## Conclusion

In summary, the results showed that galvanic corrosion strongly enhanced the corrosion of HP Mg. In addition, the *in vivo* experiments suggested accelerating corrosion of HP Mg screws when they were co-implanted with Ti screws into the SD rats’ femur, with a spacing of 5 and 10 mm. It is hypothesized that the Mg and Ti screws formed a galvanic-like cell through the blood vessels on the periosteum, which was responsible for the accelerating corrosion behavior of HP Mg. Therefore, a certain distance is critical to maintain the mechanical and biological property of Mg when it is co-implanted with Ti.

## Additional Information

**How to cite this article**: Hou, P. *et al*. Accelerating Corrosion of Pure Magnesium Co-implanted with Titanium *in Vivo. Sci. Rep.* **7**, 41924; doi: 10.1038/srep41924 (2017).

**Publisher's note:** Springer Nature remains neutral with regard to jurisdictional claims in published maps and institutional affiliations.
